# Effects of adding low-dose esketamine to sufentanil and propofol sedation during cervical conization: a single-centre, randomized controlled trial

**DOI:** 10.1186/s12871-023-02389-2

**Published:** 2024-01-04

**Authors:** Jiguo Si, Xiaomin Li, Yuqi Wang, Nianhai Feng, Min Cui

**Affiliations:** 1 Department of Anesthesiology, Zibo Central Hospital, Zibo, China; 2 Department of Anesthesiology, Zibo Maternal and Child Health Care Hospital, Zibo, China

**Keywords:** BIS-guided propofol TCI, Esketamine, Propofol, Sufentanil

## Abstract

**Background:**

Cervical conization is a brief but painful procedure that can be performed under sufficient sedation with propofol and opioids. However, this sedation approach comes with a high risk of sedation-related adverse events (SRAEs). Esketamine, an *N*-methyl-d-aspartate (NMDA) receptor antagonist, causes less cardiorespiratory depression than opioids. The aim of this study was to assess the efficacy and safety of adding a low dose of esketamine to propofol and sufentanil sedation as an opioid-reduced regimen.

**Methods:**

A total of 122 consecutive patients with ASA I-II, body mass index < 30, and STOP-BANG score < 3 who underwent cervical conization were enrolled and randomly divided into Group S and Group ES. Using a closed-loop target-controlled infusion (TCI) pump with a target bispectral index (BIS) value of 60 ± 5, patients in Group S were sedated with 0.2 mcg·kg-1 sufentanil and propofol, while patients in Group ES were sedated with 0.15 mg·kg-1 esketamine, 0.1 mcg·kg-1 sufentanil and propofol. The primary outcome was the incidence and severity of SRAEs, while the secondary outcomes included effectiveness of sedation, awakening time, psychotomimetic side effects, postoperative pain, postoperative nausea and vomiting, and patient and gynaecologist satisfaction.

**Results:**

Data from 120 patients were analysed. The incidence of composite SRAEs was significantly higher in Group S than in Group ES (85.0% vs. 56.7%, *P* < 0.05). Furthermore, the severity of SRAEs was higher in Group S than in Group ES (*P* < 0.001). There were no significant differences in the effectiveness of sedation, awakening time, psychotomimetic side effects, postoperative pain, postoperative nausea and vomiting, or patient and gynaecologist satisfaction between the two groups.

**Conclusion:**

Adding low-dose esketamine to propofol and sufentanil sedation reduces the incidence and severity of SRAEs in patients undergoing cervical conization, with equal sedation efficacy, recovery quality, and no additional psychomimetic side effects.

**Trial registration:**

ChiCTR2000040457, 28/11/2020.

**Supplementary Information:**

The online version contains supplementary material available at 10.1186/s12871-023-02389-2.

## Background

Despite efforts by the World Health Organization to eliminate cervical cancer, it remains a leading cause of morbidity and mortality in developing countries. Cervical conization is a commonly used excisional surgery for the treatment of cervical intraepithelial neoplasia (CIN) or the diagnosis of cervical cancer. As the procedure is less invasive and the operation time is usually short, this kind of surgery can be conducted with deep sedation rather than general anaesthesia with tracheal intubation or a laryngeal mask airway in clinical practice [1]. The widely used sedation protocol involves target controlled infusion (TCI) of propofol with titration of opioids [2, 3], but it was noted that the combination of propofol and opioids may lead to respiratory and hemodynamic instability. Although these cases of cardiopulmonary depression rarely pose any severe consequences under close monitoring of the administered sedation, the risk of losing airway protection, spontaneous respiration, and cardiovascular stability is always present, and the sedation care provider must be ready to manage these adverse events (AEs) throughout the procedure. Therefore, exploring a better sedation protocol to reduce the incidence of sedation-related adverse events (SRAEs) remains of paramount importance.

Esketamine is the S(+)-isomer of ketamine. As a new noncompetitive NMDA receptor antagonist, it produces both analgesic and anaesthetic effects with a lower risk of respiratory depression, and its sympathomimetic properties benefit the maintenance of stable haemodynamics during sedation. Esketamine has been proven to be three times as potent as the R(−)-isomer and twice as potent as the racemic mixture, with a significantly shorter half-life and clearance time than that of ketamine [4]. Increased potency and faster metabolism enable esketamine to produce the required analgesic and anaesthetic effects with much lower dosages, resulting in fewer undesirable psychomimetic side effects, such as dreaminess, nightmares, drowsiness, hallucinations, vertigo, and extracorporeal experiences, which are typically caused by NMDA receptor antagonists [5, 6]. A randomized controlled trial was conducted to investigate the feasibility and side effects of opioid-reduced general anaesthesia based on esketamine, and the findings indicated that esketamine provided more stable haemodynamics [7]. However, no clinical trials are available that address the SRAEs of the combination of esketamine with sufentanil as the analgesic component in propofol sedation. The aim of this study was to verify whether adding low-dose esketamine to sufentanil and propofol sedation could mitigate SRAEs, produce the same effectiveness of sedation, and generate no additional psychomimetic side effects.

## Methods

### Study design and ethics

The current study was a single-centre randomized controlled clinical trial. The study was conducted in accordance with ethical practices. Ethical approval was obtained from the Ethics Committee of Zibo Central Hospital (No. 202009001) on 01/09/2020. We registered the study at the Chinese Clinical Trial Registry on 28/11/2020 (ChiCTR2000040457). Participants received a written and oral explanation of the study and signed an informed consent form. The trial report complied with the Consolidated Standards of Reporting Trials (CONSORT) guidelines.

### Participants

A total of 122 patients scheduled for elective cervical conization between December 2020 and February 2022 were enrolled in this study. The inclusion criteria were age between 18 and 60 years, American Society of Anaesthesiologists (ASA) Physical Status I to II, body mass index (BMI) < 30 kg·m-2, and STOP-BANG score < 3. The exclusion criteria were as follows: 1) severe respiratory disease (such as asthma or pneumonia); 2) recent history of unstable angina pectoris, myocardial infarction, congestive heart failure occurring within the past 6 months, or uncontrolled hypertension; 3) central nervous system disease (such as cerebral infarction or epilepsy), increased intracranial pressure, or psychiatric disease; 4) hepatic or renal failure; and 5) known allergic reaction to any planned medication.

### Randomization and blinding

Included patients were randomly assigned to either Group S or Group ES in a 1:1 ratio using SPSS 26 software. All individuals involved in the study, including the gynaecologists, sedation practitioners, and participants, were blinded to the group allocation, except for an independent researcher who diluted 0.2 mcg·kg-1 sufentanil (for Group S) or 0.15 mg·kg-1 esketamine plus 0.1 mcg·kg-1 sufentanil (for Group ES) to the same volume as normal saline. To account for the possibility of additional injections, a double dose of these analgesics was prepared for each patient according to their group allocation.

### Sedation methods and monitoring

All patients fasted in accordance with the ASA practice guidelines for preoperative fasting [8]. Once the patient’s information was verified and intravenous access was established, standard monitoring was initiated, which included the heart rate (HR), noninvasive blood pressure (NIBP), oxygen saturation of finger pulse (SpO_2_), electrocardiograph (ECG), and respiratory rate (RR). Patients were positioned in a lithotomy position without a pillow. Oxygen was administered at a rate of 2 L·min-1 via a face mask connected to the breathing circuit of the anaesthesia machine before the onset of sedation. Then, the mainstream exhaled carbon dioxide concentration (PETCO_2_) was measured through a Philips monitor, and the tidal volume (VT) was measured through the anaesthesia machine. An assistant held the mask and ensured a secure fit of the mask on the patient’s mouth and nose, ensuring airtightness while avoiding excessive force that could make the patient uncomfortable. After sedation, a head strap was used.

Patients in both groups were sedated using the TCI of propofol with a closed-loop TCI pump (BCP-100A, Slgo, China), which could automatically adjust the targeted concentration of propofol according to the patient’s BIS value as a feedback index. The initial targeted BIS was set at 60 ± 5, the initial targeted plasma drug concentration (Cpt) was set at 2.0 mcg·mL-1, and the effect concentration (Ce) was calculated per the Marsh model. When the Ce reached 1.0 mcg·mL-1, patients in Group E received 0.2 mcg·kg-1 sufentanil, while patients in Group ES received 0.1 mcg·kg-1 sufentanil and 0.15 mg·kg-1 esketamine. When the BIS value reached 75 or lower, we started the closed loop, and the automatic control device worked itself to maintain the BIS value in a range of 60 ± 5. If the BIS value could not get below 75 with the initial Cpt or rose above 75 again during the procedure, we added the target Cpt by 0.5 mcg· mL-1 each time. Sufficient time between each adjustment was required until Ce equals Cpt. A database file, including the present BIS value, Cpt, Ce, infusion speed, and total dosage of propofol injected, could be exported and analysed from the device. These series of data were continuously written per second. The operation began when the BIS value was stably within the expected range and at least 2 minutes after the administration of analgesics. If there was body movement during the operation while the BIS value was still in the planned range, one-third of the initial dose of the same analgesic was administered.

When the oxygen saturation dropped to 90% or below, treatment for airway obstruction was performed in the following order based on severity: jaw thrust manoeuvre, insertion of a nasal airway, and insertion of a laryngeal mask. If the patient experienced apnoea, positive press ventilation was administered. Hypotension, defined as a systolic arterial pressure lower than 80 mmHg or decreased to more than 25% of baseline, was treated with 6 mg of ephedrine. Bradycardia, defined as a heart rate decrease of more than 25% of baseline, was treated with intravenous atropine 0.5 mg when the heart rate is less than 55 beats per minute.

After the completion of the operation, the TCI of propofol was stopped, and the patient’s sedation level was assessed every minute using the Modified Observed Assessment of Alertness/Sedation scale (MOAA/S). MOAA/S is a scale to describe the depth of sedation and is scored from 5 to 0: 5 = responds readily to name spoken in normal tone, 4 = lethargic response to name spoken in a normal tone, 3 = responds only after name is called loudly and/or repeatedly, 2 = responds only after mild probing or shaking, 1 = responds only after painful trapezius squeeze, and 0 = no response after painful trapezius squeeze [9]. Once the patient responded readily to her name spoken in a normal tone (MOAA/S score = 5), which was regarded as being fully awake, she was transferred to the post anaesthesia care unit (PACU) and monitored for at least 30 minutes until the modified Steward score reached 6. The Steward scoring system includes three aspects of assessment after sedation: 1) consciousness, 2 = awake, 1 = responding to stimuli, 0 = no response; 2) airway, 2 = coughing on command or crying, 1 = maintaining a good airway, 0 = airway requires maintenance; and 3) movement, 2 = moving limbs purposefully, 1 = non purposeful movement, 0 = not moving [10]. Pain and nausea were assessed in the PACU using a Visual Analog Scale (VAS) ranging from 0 to 10, with 0 regarded as no pain or nausea and 10 indicating the worst pain or nausea. When the patient’s pain VAS score was greater than 5, 30 mg of ketorolac tromethamine was administered intramuscularly.

### Outcome and outcome assessment

The primary endpoint of the study was the incidence and severity of SRAEs. The definition of the SRAEs was according to the Adverse Events Sedation Reporting tool recommended by the World Society of Intravenous Anaesthesia (World SIVA) [11]. The tool comprised both DESCRIPTION of the adverse event(s) and INTERVENTIONS performed to treat the adverse event(s). In the description section, minor risk descriptors included oxygen desaturation (75–90%) for < 60 s, nonprolonged apnoea (< 60 s), airway obstruction, bradycardia, tachycardia, hypotension, and hypertension, while sentinel risk included severe oxygen desaturation (< 75% at any time or < 90% for > 60 s) and prolonged apnoea (> 60 s). In the intervention part, minor risk included airway repositioning; moderate risk included artificial assisted ventilation, laryngeal mask airway, and oral/nasal airway; and sentinel intervention included tracheal intubation, vasopressor administration, and atropine to treat bradycardia. The severity of adverse events was defined as the most serious option checked in the tool (supt. 1).

The secondary outcomes of the study included the effectiveness of sedation, awakening time, psychotomimetic effects, postoperative pain, postoperative nausea and vomiting, and patient and gynaecologist satisfaction. The effectiveness of sedation was measured by the number of intraoperative body movements and interruptions of the surgery. Psychotomimetic side effects were assessed based on the patients’ memory of dreaming or nightmares during the sedation, drowsiness, and hallucination, which were evaluated in the PACU. Gynaecologist satisfaction with the procedure was investigated immediately after the operation, while patient satisfaction with the sedation was investigated upon their leaving the PACU and at follow-up the next day. All satisfaction degrees were measured using a Likert scoring system, with 1 = very dissatisfied and 5 = highly satisfied.

Vital signs, including HR, NIBP, SpO_2_, RR, VT and PETCO_2,_ were recorded at the beginning of sedation (T1), Ce = 1 (T2), onset of closed-loop (T3), every 5 minutes during the operation (T4), MOAA/S = 5 (T5), reaching the PACU (T6) and discharging from the PACU (T7). The BIS value, Cpt and Ce were recorded at T1-T5.

### Statistical analysis

A preliminary experiment was conducted prior to the commencement of the trial. The results of the pre-experiment revealed that the incidence of SRAEs was approximately 80% in Group E and 52% in Group ES. Based on a power of 0.90 and a significance level of 0.05, the calculated sample size using PASS 15.0 software was 55 in each group. Accounting for an estimated dropout rate of 10%, 122 patients in total were ultimately enrolled.

We conducted statistical analysis using SPSS software version 25.0 (SPSS Inc., Chicago, USA). First, we verified the normality of the measurement data with the Shapiro–Wilk test. Quantitative data with a normal distribution are expressed herein as the mean ± standard deviation, and the difference between two groups was compared using an independent-samples T test. Quantitative data with an abnormal distribution are expressed as the median [interquartile range], and the comparison between two groups was verified using the Mann–Whitney U test. Qualitative data, expressed herein as a percentage, were analysed using the Pearson chi-square test or Fisher’s exact test. Repeated measurement data were analysed using two-way repeated-measures ANOVA. A two-sided *P* value < 0.05 was considered statistically significant.

## Results

### Patient characteristics

**Fig. 1:**
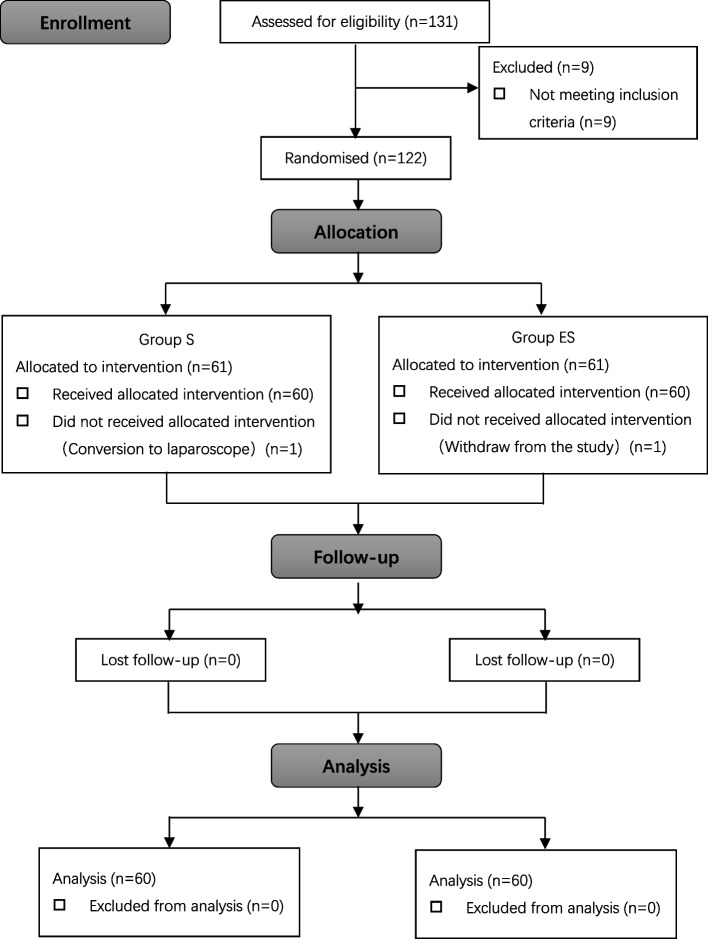
Study flow chart

From December 2020 to February 2022, a total of 122 patients were randomly allocated to Group S and Group ES according to the inclusion criteria, with 61 patients in each group. One patient in Group S did not undergo the planned procedure, and one patient in Group ES withdrew from the study. As a result, the final analysis included 60 patients in each group (Fig. 1). As listed in Table 1, there were no statistically significant differences between the two groups in terms of patient characteristics.

**Table 1:** Patients’ baseline characteristics

	Group S (*n* = 60)	Group ES (*n* = 60)	*P* value
Age (years)	42.7 ± 11.0	43.5 ± 10.2	0.687
Weight (kg)	58.4 ± 8.6	60.6 ± 8.6	0.866
Length (cm)	161.1 ± 4.5	162.1 ± 4.7	0.654
BMI (kg·m-2)	22.6 ± 3.1	23.0 ± 2.9	0.618
ASA physical status (I/II)	43/17	48/12	0.286
STOP-BANG score(0/1/2)	32/15/13	30/17/13	0.910
Smoker	3 (5.0%)	1 (1.6%)	0.619
Alcohol	4 (6.7%)	1 (1.6%)	0.364
Hypertensive disease	6 (10.0%)	3 (5.0%)	0.491
Diabetes	4 (6.7%)	1 (1.6%)	0.364
Coronary heart disease	2 (3.3%)	0 (0)	0.496
Surgery time (min)	28.7 ± 12.8	28.1 ± 13.1	0.778
Volume of intraoperative infusion (mL)	430.2 ± 101.9	400.1 ± 115.5	0.133
0.01% epinephrine cervical injected (mL)	25.7 ± 20.4	22.5 ± 13.6	0.318

Values are summarized by the mean ± standard deviation or the number (%). *ASA* American Society of Anaesthesiology, *BMI* body mass index, *CI* confidence interval

### Primary endpoint

As shown in Table 2, 51 patients in Group S and 34 patients in Group ES experienced SRAEs. The incidence of SRAEs in Group S was significantly higher than that in Group ES (85.0% vs. 56.7%, *P* < 0.05). Specifically, the incidences of respiratory SRAEs, including severe oxygen desaturation (46.7% vs. 11.7%, *P* < 0.001) or nonsevere (63.3% vs. 23.3%, *P* < 0.001), prolonged (53.3% vs. 13.3%, *P* < 0.001) or nonprolonged (73.3% vs. 28.3%, *P* < 0.001) apnoea, and airway obstruction (83.3% vs. 50.0%, *P* < 0.001), as well as the interventions performed to treat these adverse events, including airway repositioning (83.3% vs. 50.0%, *P* < 0.001), artificial assisted ventilation (60.0% vs. 16.7%, *P* < 0.001), and oral/nasal airway (36.7% vs. 13.3%, *P* < 0.05), were all significantly higher in Group S than in Group ES. Regarding cardiovascular adverse events, more patients experienced intraoperative hypotension and received ephedrine in Group S than in Group ES (28.3% vs. 13.3%, *P* < 0.05). Furthermore, there was a statistically significant difference in the severity of the adverse events between the two groups; the severity of SRAEs in Group S was higher than that in Group ES (*P* < 0.001).

**Table 2:** Incidence and severity of SRAEs

	Group S (*n* = 60)	Group ES (*n* = 60)	*P* value
Total incidence	51 (85.0%)	34 (56.7%)	0.001
Oxygen desaturation (75–90%) for 30 ~ 60 s	38 (63.3%)	14 (23.3%)	< 0.001
Oxygen desaturation (75–90%), severe (< 75% at any time) or prolonged (< 90% for> 60s)	28 (46.7%)	7 (11.7%)	< 0.001
Apnoea, not prolonged (30 ~ 60 s)	44 (73.3%)	17 (28.3%)	< 0.001
Apnoea, prolonged (> 60 s)	32 (53.3%)	8 (13.3%)	< 0.001
Airway obstruction/airway repositioning	50 (83.3%)	30 (50.0%)	< 0.001
Artificial assisted ventilation	36 (60.0%)	10 (16.7%)	< 0.001
Oral/nasal airway	22 (36.7%)	8 (13.3%)	0.003
Hypotension/ephedrine as vasopressor	17 (28.3%)	8 (13.3%)	0.043
Severity of SRAEs			< 0.001
No SRAEs	5	15	
Minimal	0	6	
Minor	18	26	
Moderate	12	8	
Sentinel	25	5	

Data are presented as the mean ± standard deviation or the number (%). *SpO*_*2*_ pulse oxygen saturation, *PETCO*_*2*_ end tidal carbon dioxide concentration, *SRAEs* sedation-related adverse events

### Secondary endpoints

Table 3 presents data on secondary endpoints. There were no differences between groups in terms of somatic motors during surgery or sedation-related interruptions of operation. No significant difference in awakening time was observed between the two groups, with a median awakening time of 7 minutes in both groups. Psychotomimetic side effects also revealed no significant difference between the two groups, with 3 patients in Group S and 8 patients in Group ES having a memory of dreaming and 1 patient in Group S reporting a bad dream during the surgery. No patient in either group experienced vertigo during the recovery time, and all patients had an orientation score of 6 upon transfer to the PACU and at discharge from the PACU. These data were constants and are therefore not presented in the table. No statistically significant difference was found in postoperative pain, additional analgesics in the PACU, postoperative nausea and vomiting during the PACU time and the next 24 hours between the two groups. The median degree of satisfaction of the patients and gynaecologists at any time point was 5, indicating high satisfaction, and no significant difference was found between the groups.

**Table 3:** Sedation effectiveness, awakening time, psychotomimetic side effects, postoperative pain, postoperative nausea and vomiting, and patient and gynaecologist satisfaction

	Group S (*n* = 60)	Group ES (*n* = 60)	*P* value
Times of somatic motors during the surgery	0 (0, 0)	0 (0, 0)	0.080
Times of sedation-related procedure interruption	0 (0, 0)	0 (0, 0)	0.139
Awakening time (min)	7 (6, 8)	7 (6, 8)	0.323
Psychotomimetic side effects			
Memory of dreaming	3 (5.0%)	8 (13.3%)	0.204
Memory of nightmares	1 (1.7%)	0	1.000
Drowsiness	1 (1.7%)	2 (3.3%)	1.000
Hallucination	0	0	
NRS of pain in the PACU	2 (1, 4)	3 (2, 5)	0.193
Additional analgesics in the PACU	8 (13.3%)	14 (23.3%)	0.239
NRS of pain over the next 24 hours	3 (2, 5)	3 (2, 5)	0.755
NRS of nausea in the PACU	0 (0, 0)	0 (0, 0)	0.319
NRS of nausea over the next 24 hours	0 (0, 0)	0 (0, 0)	0.518
Vomiting in the PACU	1 (1.7%)	0	1.000
Vomiting over the next 24 hours	6 (10.0%)	6 (10.0%)	1.000
Patient satisfaction after awakening	5 (5, 5)	5 (5, 5)	0.791
Patient satisfaction in the next 24 h	5 (5, 5)	5 (5, 5)	0.434
Gynaecologist satisfaction	5 (5, 5)	5 (5, 5)	0.574

Data are presented as the median (interquartile range) or the number (%). *NRS* numeric rating scale, *PACU* post-anaesthesia care unit

### Vital parameters during the sedation

**Fig. 2:**
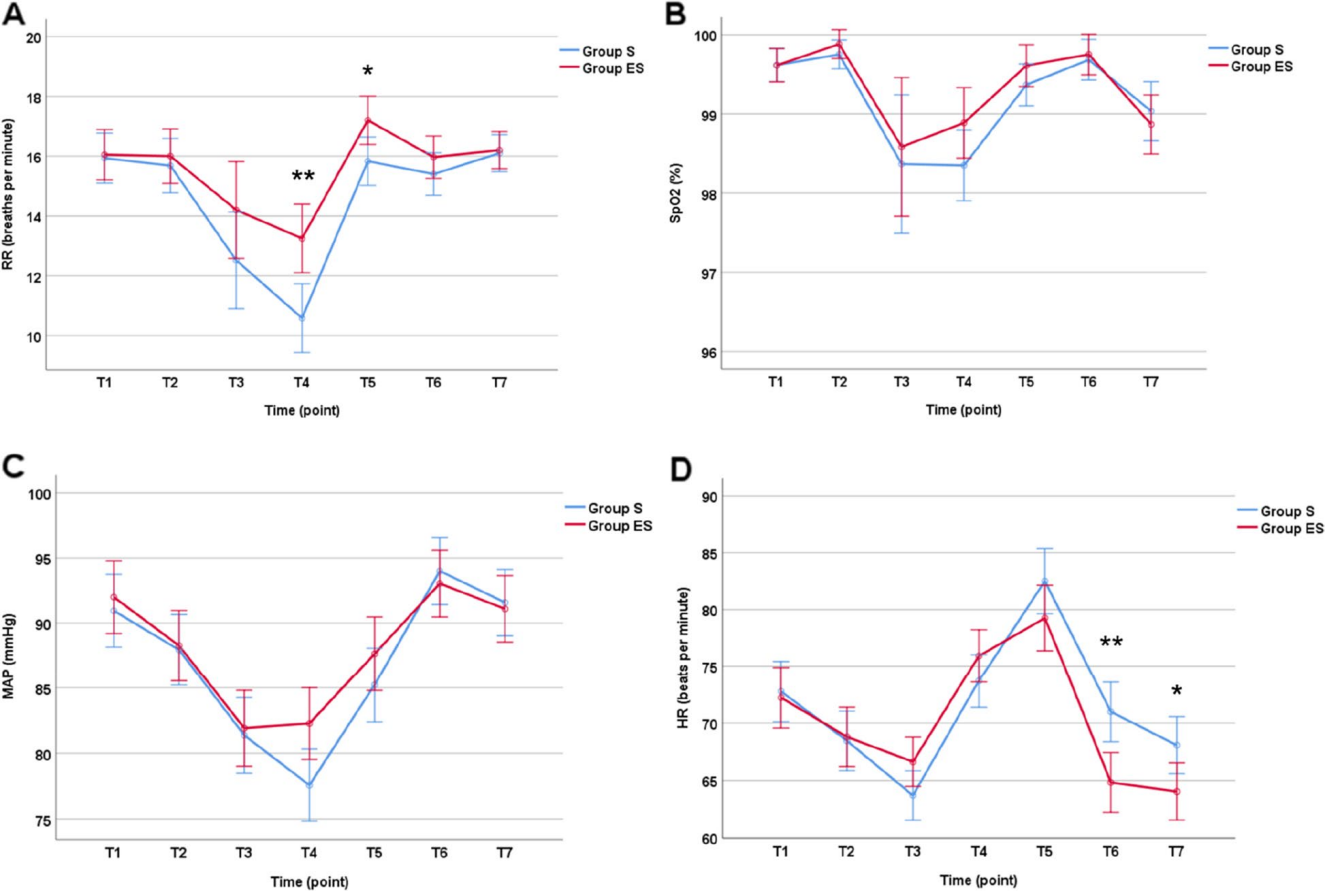
Vital signs in the perioperative period; Compared to group ES, *p < 0.05, **p < 0.01

The patients’ vital parameters during the sedation and recovery periods are presented in Fig. 2. The results showed significant RR fluctuations in both groups. In Group S, RR was significantly lower at T3 (*P* = 0.002) and T4 (*P* < 0.001) than at T1. In Group ES, the same difference was found only at T4 (*P* = 0.006). Additionally, the RR fluctuation levels at T4 and T5 were significantly lower in Group S than in Group ES (*P* = 0.001 and 0.002, respectively). The magnitude of SpO_2_ fluctuations showed no significant difference between the two groups. Compared to that at T1, SpO_2_ significantly declined at T4 in Group S (*P* < 0.001), while no such result was found in Group ES. Patients in both groups experienced a fall in mean arterial pressure (MAP) during the procedure, with patients in Group S experiencing a decline from T2 to T5 and patients in Group ES experiencing a decline from T2 to T4. The MAP of patents in Group S was significantly lower than that in Group ES at T4 (*P* < 0.001). The HR of patients in Group S was higher than that of patients in Group ES upon reaching the PACU (T6, *P* = 0.001) and being discharged from the PACU (T7, *P* = 0.025). Compared to that at T1, the HR was significantly lower at T2, T3, and T7 and higher at T5 in Group S but lower at T2, T3, T6, and T7 and higher at T5 in Group ES.

As shown in Table 4, the VT of patients in both groups decreased after sedation, with the VT of patients in Group S being significantly lower than that in Group ES at the beginning of the surgery (T3, *P* = 0.015). The minimum SpO_2_ during the entire procedure in Group S patients was significantly lower than that in Group ES patients (86.0 ± 8.6 vs. 91.6 ± 7.7, *P* < 0.001), but there was no significant difference in maximum PETCO_2_ during the entire procedure between the two groups.

**Table 4:** VT at the beginning of surgery, minimum SpO_2_, maximum PETCO_2_, total propofol and analgesic drug dose, Cpt, Ce and BIS

	Group S (*n* = 60)	Group ES (*n* = 60)	*P* value
VT at the beginning of surgery (mL)	135.0 (0.0, 226.8)	194.0 (121.5, 253.8)	0.007
Minimum SpO_2_ (%)	86.0 ± 8.6	91.6 ± 7.7	< 0.001
Maximum PETCO_2_ (mmHg)	53. 7 ± 8.6	51.1 ± 7.1	0.080
Propofol dose (mg)	372.2 ± 160.7	423.9 ± 179.2	0.099
Analgesic drug consumption (ml)	3 (3, 3)	3 (3, 3)	0.174
Cpt during the operation (mcg·mL-1)	2.8 (2.4, 3.4)	3.4 (3.0, 3.8)	< 0.001
Ce during the operation (mcg·mL-1)	2.7 (2.4, 3.1)	3.3 (2.9, 3.7)	< 0.001
Cpt at OAA/S = 5 (mcg·mL-1)	1.3 (1.0, 1.8)	1.6 (1.4, 1.9)	0.002
Ce at OAA/S = 5 (mcg·mL-1)	1.8 (1.4, 2.6)	2.3 (1.9, 2.7)	0.002
BIS at OAA/S = 5 (mcg·mL-1)	81.0 (78.0, 82.7)	78.5 (76.0, 81.0)	0.003

Data are presented as the mean ± standard deviation or the median (interquartile range). *Cpt* plasma target concentration, *Ce* effect concentration, *BIS* bispectral index

There was no statistically significant difference in the total dose of propofol and analgesic drugs between the two groups. The propofol Cpt of patients in Group S was significantly lower than that of patients in Group ES during the operation (T4, 2.8 mcg·mL-1 vs. 3.4 mcg·mL-1, *P* < 0.001) and at OAA/S = 5 (T5, 1.3 mcg·mL-1 vs. 1.6 mcg·mL-1, *P* = 0.002). The same difference was observed in Ce. Additionally, the BIS of patients in Group S was significantly higher than that of patients in Group ES when OAA/S = 5 (T5, *P* < 0.005). The above data can be found in Table 4.

## Discussion

The main objective of this randomized controlled trial was to investigate whether the addition of low-dose esketamine to propofol and sufentanil sedation could reduce the occurrence of SRAEs. The results of this study revealed that the combination of 0.15 mg·kg-1 esketamine with 0.1 mcg·kg-1 sufentanil as an analgesic component was associated with fewer and less-severe SRAEs. Moreover, this combination showed equal sedation efficacy and recovery quality with no additional psychotomimetic side effects compared to 0.2 mcg·kg-1 sufentanil as an analgesic during BIS-guided propofol sedation for cervical conization.

Esketamine, as an NMDA receptor antagonist, interacts with a number of different targets, including opioid, monoaminergic, and muscarinic receptors, as well as voltage-sensitive calcium channels [12]. It exhibits a higher receptor affinity and shorter metabolism than ketamine. In this study, the incidence and severity of SRAEs were significantly lower in Group ES, potentially due to the gentle respiratory depression properties of esketamine in comparison to opioids. Eberl S et al. assessed the effectiveness of esketamine versus alfentanil as an adjunct to propofol TCI for deep sedation during ambulant endoscopic retrograde cholangiopancreatography and found that, compared to alfentanil, low-dose esketamine reduces the total amount of propofol necessary for sedation without affecting recovery time and respiratory or cardiovascular adverse events [13]. Another study by Zhu T et al. found that administering an additional 0.2 mcg·kg-1 loading dose followed by a 0.5 mg·kg-1·h-1 infusion of esketamine provided more stable haemodynamics in opioid-reduced general anaesthesia [7]. The current study also showed a lower incidence of hypotension and a relatively higher MAP during the procedure with a combination of esketamine and sufentanil sedation. The cardiovascular stimulating properties of esketamine may have contributed to remaining hemodynamic stability during sedation. These findings are consistent with previous studies [7, 12, 14].

There is an ongoing debate about the optimal anaesthetic approach for minimally traumatic and minimally painful procedures, such as endoscopic retrograde cholangiopancreatography, transcatheter aortic valve implantation, cervical conization, and retrograde ureteral catheterization [15]. For short procedures that do not interfere with airway management, sedation without tracheal intubation has been shown to have similar safety and suitability profiles as general anaesthesia [16]. The most widely used sedation protocol involves the titration of opioids with a TCI of propofol. However, there are significant disadvantages to this regimen, particularly the risk of SRAEs, especially respiratory-related SRAEs. The sedation practitioner must intensively monitor the respiratory rate, SpO_2_, and PETCO_2_ and observe closely for apnoea or airway obstruction throughout the entire anaesthesia procedure. The use of a combination noncompetitive NMDA receptor antagonist provides a new approach for such sedation regimens. Esketamine provides both anaesthetic and analgesic effects and has fewer respiratory and circulatory depression properties than other anaesthetics and analgesics. A previous study reported that compounding low-dose esketamine could reduce opioid consumption and provide better circulatory stability in general anaesthesia [17]. However, in sedation without tracheal intubation, we are more concerned with the patient’s ability to maintain spontaneous ventilation and a patent airway. To the best of our knowledge, this is the first paper to focus on SRAEs, especially respiratory-related SRAEs, of the combination of esketamine with sufentanil and BIS-guided propofol TCI.

In this study, the sedation achieved was deeper than deep sedation with sufficient analgesics, and some would argue that it may have resulted in more cardiopulmonary side effects, especially respiratory depression. In fact, our results showed relatively higher total incidences of SRAEs: 85% in Group S and 57% in Group ES. However, minor adverse events accounted for a large proportion of these events. For example, 83.3% of patients in Group S and 50.0% of patients in Group ES experienced airway obstruction, which was managed by a simple chin lift or jaw thrust. Few of them needed a nasal airway, and no patient in either group required laryngeal or tracheal intubation. In the study, Group ES exhibited a lower total incidence of SRAEs as well as lower incidences of individual SRAEs than Group S. Additionally, the severity of SRAEs was less in Group ES than in Group S. Thus, a sedation protocol that combines a low dose of esketamine with reduced sufentanil is considered safer for patients and more convenient for anaesthesiologists.

Many previous studies have reported psychic side effects of ketamine, such as the induction of nightmares, hallucinations, extracorporeal experiences, and other psychic sensations [6, 18]; the same could happen with esketamine. However, combination with propofol or benzodiazepines can reduce these adverse events. Gruber et al. identified only one of 134 patients with hallucinations or nightmares after sedation with ketamine and midazolam. The results of the current study revealed no more memory of dreaming or bad dreams or other psychic side effects in patients sedated with a combination of esketamine and sufentanil [19]. This is in accord with the results of St Pierre and S. N. Piper [20, 21].

Daniela et al. reported that the esketamine level rapidly decreases after administration and that redosing does not compromise the recovery quality [4]. Interestingly, we found that patients sedated with a combination of esketamine fully woke up with a higher Ce, higher Cpt, and a lower BIS value. This has never been reported before, and we believe it may be related to the psychoactive effects of esketamine.

## Limitations

There were some limitations in our study. First, deep sedation is defined as a depression of consciousness during which patients cannot be easily aroused but respond purposefully following repeated or painful stimulation, while a state of losing consciousness and being unarousable even by painful stimulation should be classified as general anaesthesia. In this study, we aimed to achieve an anaesthesia level that would provide the same analgesic effects as general anaesthesia and cardiopulmonary inhibition as mild as that with deep sedation for short-duration and minimally invasive procedures. Many studies, including ours, have been helpful in exploring ways to reduce the SRAEs of deep sedation, and deep sedation has been proven to be as safe as tracheal intubation-induced general anaesthesia when performed by skilled anaesthesiologists in numerous studies [22, 23]. Second, sedation was performed using a BIS-guided regimen with a closed-loop propofol TCI. Ketamine and esketamine can trigger an increase in rapid γ-waves, resulting in an incorrect high BIS value despite deep anaesthetic levels [24, 25]. Therefore, the BIS-guided sedation regimen with esketamine may induce an unexpectedly deeper level of sedation with a greater Ce of propofol. This deeper level of sedation may counteract the positive cardiovascular effects of esketamine. However, in the current study, despite the higher Cpt and Ce of propofol in Group ES, the MAP was higher and the SRAEs were less than those in patients sedated without esketamine. This further demonstrated the advantage of adding esketamine to propofol sedation, as it induced less respiratory depression and helped maintain circulatory stability. However, quantifying the impact of esketamine on the BIS and objectively measuring the anaesthesia depth produced by different sedatives and analgesics is an interesting and worthy topic for further exploration. Third, we only assessed the psychotomimetic side effects in the PACU, and long-term psychological side effects were not evaluated. Moreover, we selected only a few indicators, such as memory of dreaming, memory of nightmares, drowsiness, and hallucination; such an assessment might be insufficient. Finally, because of concerns about the negative effects on recovery quality and psychotomimetic side effects that may be caused by esketamine, sedation with esketamine and propofol without sufentanil was not included in our study. The results of this study suggested that this compromise protocol of mixing a half dosage of both sufentanil and esketamine for propofol sedation was an appropriate regimen. Nonetheless, the safety and effectiveness of esketamine and propofol sedation without opioids deserve further study.

## Conclusion

Adding a low dose of esketamine to sufentanil and propofol sedation can reduce the incidence and severity of SRAEs in patients at average risk for SRAEs undergoing cervical conization. This sedation regimen, compared to the sedation regimen with sufentanil and propofol, provides equal sedation efficacy, recovery quality, and patient and gynaecologist satisfaction without additional psychomimetic side effects. The compromise protocol of administering a half dosage of both sufentanil and esketamine during BIS-guided propofol TCI sedation is an appropriate regimen.

### Supplementary Information

**Additional file 1.**

## Data Availability

The datasets supporting the conclusions of this article are available in the Clinical Trial Management Public Platform, http://www.medresman.org.cn/uc/project/projectedit.aspx?proj=7307. The datasets analysed during the current study are available from the corresponding author upon reasonable request.

## Abbreviations

SRAEs: Sedation-related adverse events
NMDA: N-methyl-d-aspartate
TCI: Target-controlled infusion
BIS: Bispectral index
CIN: Cervical intraepithelial neoplasia
BMI: Body mass index
ASA: American Society of Anesthesiologists
HR: Heart rate
SpO_2_: Pulse oxygen saturation
RR: Respiratory rate
PETCO_2_: End-tidal carbon dioxide concentration
VT: Tidal volume
MAP: Mean arterial pressure
Cpt: Target plasma concentration
Ce: Effect site concentration
MOAA/S: Modified observed assessment of alertness/sedation
PACU: Postanaesthesia care unit
