# Supplementary material for: Effects of adding low-dose esketamine to sufentanil and propofol sedation during cervical conization: a single-centre, randomized controlled trial
Source: BMC Anesthesiol. 2024 Jan 4;24:15. doi: 10.1186/s12871-023-02389-2 (PMC10765731; doi:10.1186/s12871-023-02389-2)
Supplement: Supplementary file 1 — Additional file 1. [file 12871_2023_2389_MOESM1_ESM.pdf]

World SIVA adverse sedation event reporting toll

Step 1: Was there one or more adverse events associated with this sedation encounter?

☐ No, this form is now complete. ☐ Yes, fill out remainder of form below.

Step 2: Please DESCRIBE the event(s). Check all that apply.

Minimal risk descriptors	Minor risk descriptors	Sentinel risk descriptors
☐Vomiting/Retching	☐Oxygen desaturation(75-90%) for 30~60s	☐Oxygen desaturation, severe(<75% at any time) or prolonged(<90% for > 60s)
☐Subclinical respiratory depression	☐Apnoea, not prolonged (30~60s)	☐Apnoea, prolonged(>60s)
☐Muscle rigidity, myoclonus	☐Airway obstruction	☐Cardiovascular collapse/ shock
☐Hypersalivation	☐Failed sedation	☐Cardiovascular arrest/ absent pulse
☐Paradoxical response	☐All ergic reaction without anaphylaxis	
☐Recovery agitation	☐Bradycardia （> 25%Baseline）	
☐Prolonged recovery	☐Tachycardia （> 25%Baseline）	
	☐Hypotension （> 25%Baseline）	
	☐Hypertension （> 25%Baseline）	
	☐Seizure	

Step 3: Please note the INTERVENTIONS performed to treat the adverse event(s). Check all the apply.

Minimal risk	Minor risk	Moderate risk	Sentinel intervention
☐No intervention performed	☐Airway repositioning	☐Bag valve mask-assisted ventilation	☐Chest compressions
Administration of:	☐Tactile stimulation	☐Laryngeal airway	☐Tracheal intubation
☐Additional sedatives	Administration of:	☐Oral/nasal airway	Administration of:
☐Antiemetic	☐Supplemental oxygen, new or increased	☐CPAP	☐Neuromuscular block
☐Antihistamine	☐Antisialogogue	Administration of:	☐Pressor/ epinephrin：
		☐Reversal agents	☐Atropine to treat bradycardia
		☐Rapid i.v. fluids	
		☐Anticonvulsant i.v.	

Step 4: Please note the OUTCOME of the adverse event (s). Check all the apply.

Minimal risk outcome	Moderate risk outcome	Sentinel outcome
☐No adverse outcome	☐Unplanned hospitalization or escalation of care	☐Death
		☐Permanent neurological deficit
		☐Pulmonary aspiration syndrome

Step 5: Assign a SEVERITY rating to the adverse event(s) associated with this sedation encounter.

☐Sentinel ☐Moderate ☐Minor ☐Minimal

Docter：______________
